# Tumours of the Groin and Their Differential Diagnosis

**Published:** 1896-07-11

**Authors:** W. Ernest Miles

**Affiliations:** House Surgeon to St. Mark's Hospital for Diseases of the Rectum; late House Surgeon to the Metropolitan Hospital, and to the Radcliffe Infirmary, Oxford


### Medical Progress and Hospital Clinics.

[*The Editor will be glad to receive offers of co-operation and contributions from members of the profession. All letters should be addressed to* The Editor, at the Office, 428, Strand, London, W.C.]

### TUMOURS OF THE GROIN AND THEIR DIFFERENTIAL DIAGNOSIS.

By W. Ernest Miles, F.R.C.S.Eng., House Surgeon to St. Mark's Hospital for Diseases of the Rectum; late House Surgeon to the Metropolitan Hospital, and to the Radcliffe Infirmary, Oxford.

(*Continued from page* 225.)

We will consider these diseased states under two headings: (*a*) Inflammatory, whether arising from local infection or from constitutional states, such as syphilis or tuberculous disease; (*b*) malignant disease, either primary or secondary. Before detailing the special features of each of these diseased conditions it might be as well to point out that any tumour may be considered to be of glandular lymphatic origin if it presents the following signs, namely, if it occurs in the site of a known group of glands and present the ovoid or globular outline of these bodies; if there is more than one; if it is movable under the skin and over the underlying structures, and especially if there is some obvious exciting cause, either local or constitutional.

(*a*) Of inflammatory origin.

(1) *Simple adenitis.*—Inflammation of a gland is produced through receiving lymph from some sore or abrasion situated in the region from which it obtains its supply. If the gland be painful, distinctly enlarged, tender, more or less fixed in the surrounding tissue; if the skin over it is red or œdematous, and especially if some apparent local source of infection is detected, the nature of the swelling is at once evident. An inflamed gland often goes on to suppuration, and nearly always does so when lymph is conveyed to it from a local contagious ulcer (soft chancre) on the penis and is then known as a suppurating bubo.

Occasionally a gland in the groin is found enlarged, painful, and tender, and yet no source of infection can be detected. In these cases a history of a strain is obtained, to which the grandular enlargement is ascribed. I do not know of any explanation of the reason for this, but I myself am of opinion that the enlargement is brought about in this wise. In all cases of strain there is undue stretching of the parts, and I believe that as a result of this over-stretching some of the efferent lymphatic vessels are torn at the point where they emerge from the gland. The effect of this is, that on the one hand more lymph reaches the gland than escapes from it, whereby engorgement ensues; and on the other lymph, and perhaps a small quantity of blood, is extravasated in the tissues round the gland; these two conditions combined occasion the enlargement alluded to.

(2) *Syphilitic adenitis.*—As a result of syphilis enlargement of glands takes place. If the affected glands are multiple, hard and shot-like to the touch, freely movable in the surrounding tissues, neither painful nor tender, and especially if other groups of glands in the body are similarly affected, and also evidence of other syphilitic lesions is forthcoming, syphilitic adenitis may be safely diagnosed.

(3) *Tuberculous adenitis* is a very common affection. If the enlargement is chronic, slowly progressing from gland to gland, the glands remaining at first discrete, and subsequently becoming matted together in a mass; if the swellings are firm, rounded in outline and painless, and especially if they show signs of softening, and the softened parts fluctuate, the disease may be considered to be of a tuberculous nature.

(*b*) Of a malignant nature.

(1) *Carcinomatous.*—Carcinoma never affects glands primarily. When glands are the seat of carcinomatous deposit, a primary growth is certain to be found in the region from which they receive lymph. At first they are simply enlarged, retain the ovoid shape of a healthy gland, are very hard and indurated, and move freely in the surrounding tissues. Later on they show evidence of infiltrating their surroundings, become adherent to both skin and underlying structures, and progressively fuse together in a mass. In the groin, the horizontal set is the one most commonly affected, the primary growth being found either on the penis or scrotum in the male, or on the labium in the female. If, then, the glands of the groin, particularly those of the horizontal set, rapidly increase in size, become adherent to skin and underlying tissues, and are very hard and indurated, they are probably carcinomatous, and a search will in all cases reveal a primary carcinomatous growth.

(2) *Sarcomatous.*—Sarcoma occurs in lymphatic glands of the groin most commonly as primary growths. When the disease is secondary, it is of a melanotic nature, and follows a primary melanotic sarcoma arising somewhere in the region whence these glands obtain their lymph. Not long ago I saw a large, rapidly growing tumour of the femoral glands, supervening upon a primary melanotic sarcoma situated in the skin over the internal condyle of the femur. When sarcoma occurs primarily in lymphatic glands, it is usually of the small round-celled type, and is known as lymphosarcoma, or lymphoma. This disease is, as a rule, confined to one group of glands, one or more individual glands being affected. It occurs as rounded, soft tumours, rapidly increasing in size, and showing a marked tendency to infiltrate surrounding structures. The skin soon becomes adherent and ulcerates, the growth then becoming a fungating mass, which bleeds readily. Not uncommonly soft fluctuating spots occur in different parts of the growth, indicating the occurrence of hæmorrhages within its substance. Occasionally the growth pulsates distinctly, and may simulate aneurism. The diagnosis will be arrived at by noting the rapid growth of the tumour, its distinct infiltrating character, the occurrence of hæmorrhages, and especially by the early dissemination of the disease in distant parts, such as the lungs and liver. The absence of a primary growth will distinguish lymphoma from carcinoma or melanosis. There is, however, one condition with which it may be confounded, and that is the enlargement of glands that takes place in Hodgkin's disease, or, as it is sometimes called, lymphadenoma. In this disease several groups of glands are usually involved at the same time, especially those of the neck and axilla. In the same group several glands are affected, forming firm, painless, slowly growing tumours, which show no tendency to soften or caseate. At first the affected glands have the appearance of being simply hypertrophied, and remain quite distinct from one another, but later on they may fuse, the resulting mass presenting a lobulated appearance. The diagnosis of this disease is based upon the fact that several groups of glands are simultaneously affected in different parts of the body; the course is progressive though slow, the glands do not break down or soften, the spleen is almost always found to be enlarged, and that there is progressive anæmia. An examination of the blood will reveal little or no relative increase of leucocytes, but marked diminution in the number of red corpuscles.

Before leaving the subject of swellings arising in connection with lymphatic glands, it is necessary to point out that, as a result of inflammation, these bodies may break down and form collections of pus or abscesses. These abscesses, which are situated in the subcutaneous tissue, may be acute or chronic.

*Acute abscess*.—If the swelling is circumscribed and fluctuating, and attended with redness, heat, pain of a throbbing character, great tenderness, and pyrexia, an acute abscess is to be diagnosed.

*Chronic abscess* forms a localised fluctuating swelling, unattended by the signs of acute inflammation. The skin and surrounding tissues are adherent, and the swelling is not movable upon the underlying structures. It is distinguished from a cyst by being less tense, and not so globular in shape.

Thus far we have considered all the tumours that may arise in the superficial structures of the groin, namely, the skin and subcutaneous connective tissue and fat. I may mention, however, that there is yet another tumour, which is sometimes, though very rarely, met with in this locality, having a developmental origin—I refer to an accessory mammary gland. Readers will remember that in the lower animals the abdominal mammary glands are arranged in two parallel sets, one on either side of the linea alba. They receive their blood supply from the long arterial anastomosis between the superior epigastric branch of the internal mammary artery and the deep epigastric branch of the external iliac. Accordingly, when accessory mammary glands occur abdominally in the human subject, they are located somewhere along this anastomotic line, and when met with in the groin are always situated above Poupart's ligament. They vary in size, are always congenital, and form circumscribed, soft, minutely lobulated tumours, having no tendency to increase materially in size, and are freely movable under the skin and upon the underlying structures. The diagnosis may be based upon the above features, and if some sort of a rudimentary nipple is discernible, and, moreover, if the gland enlarges during menstruation, no difficulty will be experienced in distinguishing the nature of the growth from a congenital hygroma. In another paper I shall deal with those tumours that originate in, or come into relation with, the deeper structures of the groin.

